# Uptake of COVID-19 vaccine among female healthcare workers in Syria: results from a 2022 cross-sectional survey

**DOI:** 10.1186/s13031-025-00700-1

**Published:** 2025-08-18

**Authors:** Zlatko Nikoloski, Elnur Aliyev, Robert Bain, Leonardo Menchini, Sahar Hegazi, Mai Zalkha, Shaza Mouawad, Neha Kapil, Amaya Gillespie

**Affiliations:** 1 LSE, London, UK; 2 UNICEF, Country Office, Istanbul, Turkey; 3 UNICEF, MENA Regional Office, Amman, Jordan; 4 UNICEF, Country Office, Amman, Jordan; 5 UNICEF, Country Office, Damascus, Syria; 6 UNICEF, New York, NY USA; 7 UNICEF Regional Office, Amman, Jordan

**Keywords:** Vaccines, Syria, Covid, Vaccine uptake

## Abstract

**Background:**

Healthcare workers play an important role in administering COVID-19 vaccines, particularly in conflict-affected settings. Syria has endured a protracted conflict for over a decade and while most of the healthcare workers in the country have been vaccinated with at least one COVID-19 vaccine dose, vaccinating all of them would reduce their risk of COVID-19 complications, given their daily interactions with patients.

**Methods:**

The goal of this study was to better understand the main barriers to uptake of COVID-19 vaccines among female healthcare workers in Syria. Using data from a wider national survey of 17,000 respondents conducted between October and November 2022, we analysed a sub-sample of 4136 responses from female healthcare workers, across 14 Governorates. The main outcome of interest was vaccination status, (vaccinated, willing (but not yet vaccinated), unsure about vaccination and finally, those unwilling to receive a COVID-19 vaccine). We present descriptive information about the sample and conduct a multivariate logistic regression analysis to shed light on some of the barriers preventing COVID-19 vaccination uptake.

**Results:**

We find that the vast majority (93.7%) of female healthcare workers have received at least one COVID-19 vaccination dose. We find that attitudes and beliefs around COVID-19 vaccines impact upon the decision to get a vaccination—positive attitudes around effectiveness and safety of the vaccines increase the likelihood of being vaccinated or willing to be vaccinated. More specifically, healthcare workers which believe in the safety of the vaccines are twice as likely to get vaccinated relative to those who don’t. By contrast, we find that neutral attitudes regarding the vaccines are associated with vaccine indecision among female healthcare workers. In addition, we also find that female healthcare workers tend to trust COVID-19 vaccine information from their peers—close to 99% of vaccinated female healthcare workers tend to trust the vaccine information received from their peers.

**Implications:**

While the vaccination rates among healthcare workers are high, the results could further help in devising strategies for tackling the structural and individual barriers towards vaccine uptake among healthcare workers.

**Supplementary Information:**

The online version contains supplementary material available at 10.1186/s13031-025-00700-1.

## Introduction and literature review

The rollout of the COVID-19 vaccination programmes (albeit with different speed and intensity across different countries), helped significantly in the process of reigning in the virus, allowing life to resume as before. However, while most of the adult population across the globe has been COVID-19 vaccinated (at least once), pockets of vaccine resistance exist, particularly in the low and middle income countries [1, 2]. Vaccinating frontline healthcare workers is of particular importance. While healthcare workers have been previously shown to be among the most highly vaccinated, they are also among the higher risk for exposure to the Corona Virus and encountering vulnerable populations [1]. Thus, they are a priority group when it comes to COVID-19 vaccines. To devise the appropriate strategies to approach individuals who are vaccine hesitant, further understanding of the reasons for the stated vaccine hesitancy among healthcare workers is needed.

### Syrian context

Syria has gone through a tremendous political and security transition over the last few months. In December 2024, Hayat Tahrir al-Sham (HTS), a UN-designated terrorist group that previously governed Idlib in northwest Syria, captured the Syrian capital, Damascus, and declared a transitional government. The transitional government was replaced in April 2025, and a new constitutional declaration was also issued. While HTS has been dissolved, its members retain significant leadership positions [3]. While Syria entered a new era in 2025, the humanitarian crisis is far from over. Continuous hostilities in the northern and southern parts of the country, and recently in the coastal area, continue to trigger additional needs among the population, including displacement and protection concerns, as well as hindering humanitarian operations and access [4].

The economic situation has deteriorated across Syria, previously categorised as a middle-income country, and the price of basic goods is high [5]. Eighty per cent of the population now live below the international poverty line [6]. The country’s demographic profile has also changed as a result of the conflict. Many men between the ages of 15–45 have been killed, conscripted, are in hiding or migrating to find work, resulting in a high number of female-headed households [7]. Given the hostilities in the country and the fact that healthcare facilities have sometimes been targeted, the official number of healthcare workers in the country is not known [8].

### Literature review

Over the last couple of years, a solid body of evidence across the Eastern Mediterranean region has emerged which has looked at vaccination of healthcare workers. Overall, the general finding that emerges from these studies is that the prevalence of vaccination rates among healthcare workers is high. The studies in the region have been done in: Sudan [9], Iraq [9], Egypt [10] as well as Morocco [11]. The studies revealed that better confidence in COVID-19 related information was associated with higher probability of being vaccinated against COVID-19. While most studies on healthcare workers suggest high COVID-19 acceptance rate, a handful of studies have also documented hesitancy related to COVID-19 vaccinations. Fears around low effectiveness as well as unwanted side effects of the vaccine have been listed as the main reason for COVID-19 vaccine hesitancy among healthcare workers [11, 12]. Most recently, a systematic review of available studies have also documented the vaccine hesitancy among healthcare workers in the Arab countries [13] (although the study includes significant number of studies from the high income countries in the region and also focuses on a broader period also including the time before COVID-19 vaccines were introduced).

In addition to healthcare workers, a body of evidence across the Eastern Mediterranean region has emerged that has analysed the gender dimension of the COVID-19 vaccination campaigns. Studies have found that women are more vaccine hesitant than men [14, 15]. The explanation for these gender differences have included: (i) men having to spend more time outside of the house (e.g. for work) and thus they may feel a higher need to be vaccinated,(ii) men having better access to health; as well as (iii) overall susceptibility to false beliefs and conspiracy theories among women [15–17].

However, there is very little available evidence about the vaccination status of the female healthcare workers, particularly in the context of Syria. With that said, in this paper we analyse the willingness of female healthcare workers to take up the COVID-19 vaccine, while focusing on some of the main correlates to vaccine uptake.

## Methodology

### Design and ethical approval

A COVID-19 KAP (knowledge, attitude and practices) survey was administered towards the end of 2022 on the entire sample of 17,000 individuals. The Health Media Lab (HML) Institutional Review Board (IRB) provided ethical review approval for the study (HML IRB Review #380SYRI21). Each respondent’s participation in the study was voluntary and they signed an informed consent prior to being enrolled in the study.

### Sampling

The sampling procedure followed convenience sampling of available healthcare facilities, so no power calculations were conducted prior to embarking upon the data collection. Thus, the sample is not representative on national or subnational level. All of the participants in the study were randomly selected from the sample of healthcare facilities and the data collection was done face-to-face, using a previously tested survey instrument.

This study is based on a sub-sample of 4136 female healthcare workers drawn from the wider sample mentioned above. These include respondents who are trained as female healthcare workers but also auxiliary staff (as we further report in the results section below). So, in other words, our sample includes both, auxiliary medical staff as well as trained medical professionals.

### Instrument

The instrument was tested during the pilot for optimal length. It was conducted in Arabic by both, female and male interviewers who entered the data. This means that each respondent was surveyed face-to-face. The development of the questions was based on qualitative studies done with communities and providers, although they were not included in the study per se. These included consultations on what types of questions to include and how to word them. The questionnaire was not divided into different sections, but all questions were asked in one sequence.

### Variables

In conducting the analysis, we decided to follow an established approach of distilling main demographic and socio-economic characteristics of four vaccination personas. Four vaccination personas were distilled based on the responses provided: vaccinated, willing (but not yet vaccinated), unsure about vaccination and finally, those unwilling to receive a COVID-19 vaccine. They were essentially based on two questions from the survey: (a) have you been vaccinated against COVID-19; and (b) are you willing to get vaccinated? Based on these two questions were have derived the four vaccination personas mentioned above. All of the stated variables are binary (0–1) and were used as dependent variables in the statistical analysis further described below.

In studying the characteristics of the personas, three main blocks of variables were defined: (a) demographic (e.g. gender, age); (b) attitudes towards vaccines (e.g. beliefs in the benefits that vaccines bring to the family and the community, effectiveness of the vaccines and fear of side effects, all measured on a numerical Likert scale from 1 to 5); and (c) platforms that could be used to increase the COVID-19 vaccination coverage (e.g. source of information). These three blocks of variables represent the independent variables in our analysis.

### Statistical methods

In doing the analysis we followed a descriptive statistics approach (i.e. cross tabulations across each persona and the set of independent variables mentioned above; p-value of the Chi^2^ test was also reported to attest to the strength and significance of the associations). In addition, and as a robustness check, we have added a logistic model on correlates of vaccination status (for the vaccinated healthcare workers only). A final robustness check involved questions on routine immunization (although it’s important to note that these questions were asked only on a subset of respondents, roughly 40 percent of all respondents). In addition, there were only two vaccination personas that could be distilled from these questions: vaccinated and not vaccinated. The dependent variable was being vaccinated (0–1) which was regressed on the set of independent variables mentioned above. All analyses were done in Stata 18.5.

## Results

Table 1 captures the basic descriptive statistics of the sub-sample used in the analysis. Most of the female healthcare workers (96.0%) are between the age of 18 and 55. Given the nature of our sample (which includes both, medically trained professionals and auxiliary staff), about a third of the sample has a university degree. Furthermore, 43.4 percent of the respondents in the sample have completed a secondary school. The majority of them (59.8%) trust all vaccines. In addition, the share of female healthcare workers who is vaccinated is extremely high at 93.7%, with another 1.3% willing to be vaccinated. Only 3.1% of them are not willing to be vaccinated. In addition, we provide a breakdown of the sample by governorates. As evidenced by the table, about a quarter of the respondents are from Homs. While there are respondents across all of the country’s governorates, the majority of respondents are concentrated in four main governorates: Homs, Hama, Lattakia and R Damascus. In the rest of this section, we further elaborate on the findings related to the four vaccination personas among female healthcare workers.

**Table 1:** Syria COVID-19 vaccination status survey—female healthcare workers

	%	N
*Age*		
18–55	96.0	3969
55 or more	4.0	167
*Education level*		
Preparatory	24.5	1013
Primary	2.5	102
Secondary	43.4	1794
University and above	29.7	1227
*Trust in the vaccines*		
I do not trust vaccines	4.6	176
I only trust a few vaccines	35.6	1375
I trust all vaccines	59.8	2309
*Vaccination status*		
Vaccinated	93.7	3829
Not vaccinated but willing	1.3	54
Not vaccinated and undecided	2.0	80
Not vaccinated and unwilling	3.1	125
*Governorate*		
Al-Hasakeh	1.7	70
Aleppo	1.4	56
Ar-Raqqa	0.6	23
As-Sweida	7.4	305
Damascus	0.8	32
Dar'a	0.7	27
Deir-ez-Zor	3.4	140
Hama	18.3	755
Homs	23.6	975
Idleb	0.5	21
Lattakia	14.5	600
Quneitra	4.6	191
R. Damascus	14.7	608
Tartous	8.1	333

### Vaccinated

This vaccination persona is characterised by a few main characteristics. First, this vaccination persona tends to be very well educated. Moreover, this vaccination persona is more informed about COVID-19. More specifically, 98.3% of female healthcare workers who reported receiving information all the time, are also vaccinated (Table 3). Third and most importantly, this vaccination persona tends to have positive attitudes and beliefs around the vaccines. Over 90% of those vaccinated are also convinced that the vaccines are safe and important to health (Table 3). In addition, this vaccination persona tends to trust their colleagues the most. More specifically, 98.6% of female healthcare workers with robust trust in healthcare workers are vaccinated. Finally, this vaccination persona tends to also trust all vaccines. 98.3% of vaccinated tend to trust all vaccines. 94.8% believe in the fairness of the distribution of vaccines and 97.3% believe in the importance of the vaccines for one’s health. Importantly, as Table 3 suggests, this vaccination persona tends to encourage others to take up the vaccine; and, more importantly, this vaccination persona is associated with people with similar beliefs. Furthermore, as Table 4 indicates, there is no statistically significant link between this persona and the most trusted source of information. A formal modelling exercise conducted on a set of socio-demographic and beliefs correlates of being vaccinated has yielded similar results (please see Table A1).

### Not vaccinated but willing

As in the vaccination persona above, here as well, we find a robust link between trust in healthcare workers and willingness to vaccinate. More specifically, 89% of those willing to be vaccinated have listed healthcare workers as the most trusted source for COVID-19 related information (furthermore, as demonstrated by Table 3, 3.6% of respondents with high trust in healthcare workers are willing to be vaccinated, as opposed to none among those with little or no trust in healthcare workers). In this vaccination persona, however, the positive beliefs around COVID-19 vaccines tend to play a lesser role. Still, this persona believes in the protection that the vaccines provide to the family and the community.

This persona does not seem to be concerned about fairness in the distribution of vaccines. They may have some concerns about vaccine safety but tend to trust vaccines in general and tends to recognise vaccines as important. As in the case of vaccinated, this vaccination persona tends to spend time with people with similar belief system. Table 4 lists some of the factors associated with not trusting vaccines and it suggests that among this vaccination persona, confusing information is the predominant one (40.7%). Furthermore, Table 4 suggests self-reported factors that may increase the willingness of this persona to be vaccinated, including, receiving more information on the side effects (40.7% of respondents) as well as on the safety of the vaccines (27.8%).

### Not vaccinated and undecided

Table 2 reveals that there is no clear link between education attainment and this vaccination persona. This vaccination persona tends to receive very little information about COVID-19 (4.8% of respondents who never receive news are undecided, compared to 0.3% who receive news about COVID-19 all the time). As evidenced by Table 3, this vaccination persona tends to be neutral about COVID-19 attitudes and beliefs. In other words, a higher share of female healthcare workers with neutral beliefs are undecided regarding obtaining a COVID-19 vaccine. For example, 6.9% of those with neutral beliefs around the safety of the vaccines are also undecided regarding the vaccine (similarly, 4.1% of those with neutral beliefs about how challenging is to get the vaccine are undecided). This group also tends to be neutral about whether vaccines offer protection to family and community.

**Table 2:** Demographic characteristics and vaccination status, female healthcare workers

	Vaccinated n (%)	Willing n (%)	Undecided n (%)	Not willing n (%)	Total (n)
*Age*					
18–55	3675 (93.6)	52 (1.3)	77 (2.0)	121 (3.1)	3925
Over 55	154 (94.5)	2 (1.2)	3 (1.8)	4 (2.5)	163
*Education*					
Preparatory	925 (92.6)	18 (1.8)	23 (2.3)	33 (3.3)	999
Primary	92 (92.9)	3 (3.0)	2 (2.0)	2 (2.0)	99
Secondary	1662 (93.4)	22 (1.2)	36 (2.0)	59 (3.3)	1779
University and above	1150 (95.0)	11 (0.9)	19 (1.6)	31 (2.6)	1211

**Table 3:** Beliefs and attitudes towards the COVID-19 vaccines and vaccination status, female healthcare workers

**How often do you receive information about Covid-19?**						
	Vaccinated (%)	Willing (%)	Undecided (%)	Not willing (%)	Total(n)	p-value
Never	207 (83.5)	4 (1.6)	12 (4.8)	25 (10.1)	248	< 0.001
Sometimes	2125 (92.8)	34 (1.5)	59 (2.6)	72 (3.1)	2290	
Often	1100 (96.0)	14 (1.2)	8 (0.7)	24 (2.1)	1146	
All the time	397 (98.3)	2 (0.5)	1 (0.3)	4 (1.0)	404	
**How challenging is to get the Covid-19 vaccine**						
1	2427 (93.2)	36 (1.4)	53 (2.0)	89 (3.4)	2605	< 0.001
2	404 (94.0)	4 (0.9)	5 (1.2)	17 (4.0)	430	
3	338 (92.1)	5 (1.4)	15 (4.1)	9 (2.5)	367	
4	153 (89.0)	8 (4.7)	5 (2.9)	6 (3.5)	172	
5	507 (98.6)	1 (0.2)	2 (0.4)	4 (0.8)	514	
**How safe do you think the Covid vaccines are**						
1	196 (83.1)	0 (0.0)	3 (1.3)	37 (15.7)	236	< 0.001
2	155 (72.1)	3 (1.4)	14 (6.5)	43 (20.0)	215	
3	658 (87.5)	11 (1.5)	52 (6.9)	31 (4.1)	752	
4	812 (96.3)	18 (2.1)	8 (1.0)	5 (0.6)	843	
5	2008 (98.3)	22 (1.1)	3 (0.2)	9 (0.4)	2042	
**Do you think you are at risk when taking the Covid vaccines**						
1	213 (80.7)	5 (1.9)	3 (1.1)	43 (16.3)	264	< 0.001
2	191 (74.9)	1 (0.4)	19 (7.5)	44 (17.3)	255	
3	769 (90.4)	10 (1.2)	46 (5.4)	26 (3.1)	851	
4	726 (95.3)	24 (3.2)	7 (0.9)	5 (0.7)	762	
5	1930 (98.7)	14 (0.7)	5 (0.3)	7 (0.4)	1956	
					0	
**Trust in the Covid-19 vaccines**					0	
					0	
I do not trust vaccines	110 (65.9)	2 (1.2)	11 (6.6)	44 (26.4)	167	< 0.001
I only trust a few vaccines	1259 (93.1)	22 (1.6)	38 (2.8)	33 (2.4)	1352	
I trust all vaccines	2261 (98.3)	17 (0.7)	12 (0.5)	11 (0.5)	2301	
**Trust in the healthcare workers**						
1	3 (60.0)	0 (0.0)	0 (0.0)	2 (40.0)	5	< 0.001
2	2 (100.0)	0 (0.0)	0 (0.0)	0 (0.0)	2	
3	40 (85.1)	91 (2.1)	5 (10.6)	1 (2.1)	137	
4	155 (93.9)	6 (3.6)	0 (0.0)	4 (2.4)	165	
5	708 (98.6)	5 (0.7)	1 (0.1)	4 (0.6)	718	
**Fairness in distribution of the vaccines**						
1	166 (90.2)	3 (1.6)	4 (2.2)	11 (6.0)	184	< 0.001
2	181 (91.0)	2 (1.0)	4 (2.0)	12 (6.0)	199	
3	467 (91.4)	4 (0.8)	17 (3.3)	23 (4.5)	511	
4	634 (93.0)	16 (2.4)	8 (1.2)	24 (3.5)	682	
5	2391 (94.8)	29 (1.6)	47 (1.9)	55 (2.2)	2522	
**Importance of the vaccines for one's health**						
1	143 (80.8)	4 (2.3)	2 (1.1)	28 (15.8)	177	< 0.001
2	114 (73.1)	0 (0.0)	5 (3.2)	37 (23.7)	156	
3	541 (88.5)	7 (1.2)	33 (5.4)	30 (4.9)	611	
4	737 (93.8)	25 (3.2)	13 (1.7)	11 (1.4)	786	
5	2294 (97.3)	18 (0.8)	27 (1.2)	19 (0.8)	2358	
**Protection offered to family and community**						
1	129 (74.6)	3 (1.7)	1 (0.6)	40 (23.1)	173	< 0.001
2	101 (63.0)	1 (0.6)	7 (4.2)	53 (32.1)	162	
3	480 (83.8)	6 (1.1)	59 (10.3)	28 (4.9)	573	
4	922 (96.1)	26 (2.7)	9 (0.9)	2 (0.2)	959	
5	2194 (98.9)	18 (0.8)	4 (0.2)	2 (0.1)	2218	
**Have concerns about the vaccine**						
No	3257 (98.0)	23 (0.7)	18 (0.5)	27 (0.8)	3325	< 0.001
Yes	572 (75.0)	31 (4.1)	62 (8.1)	98 (12.8)	763	
**Encouraged to take the vaccine**						
No	57 (33.7)	13 (7.7)	27 (16.0)	72 (42.6)	169	< 0.001
Yes	3597 (99.0)	17 (0.5)	10 (0.3)	9 (0.3)	3633	
**Did you encourage others to take the vaccine**						
No	579 (79.8)	21 (2.9)	46 (6.3)	80 (11.0)	726	< 0.001
Yes	2914 (98.6)	21 (0.7)	11 (0.4)	11 (0.4)	2957	
**Do you know people that are not vaccinated**						
No one	130 (97.7)	2 (1.5)	0 (0.0)	1 (0.8)	133	< 0.001
A very few	1841 (97.7)	10 (0.5)	13 (0.7)	20 (1.0)	1884	
A lot	558 (83.7)	17 (2.6)	25 (3.8)	67 (10.0)	667	

Those who are undecided tend to only trust a few vaccines. While this persona trusts their colleagues, they tend to trust them less than the two personas above. As evidenced by Table 3, 10.6% of those with neutral predisposition towards their colleagues have stated that they are undecided. Furthermore, this vaccination persona hasn’t been encouraged to be vaccinated, and it does not encourage others to take the vaccine. Similar to those willing to be vaccinated, this vaccination persona lists receiving confusing information (48.8%) as the main reason for not trusting the vaccines, as further corroborated by Table 4. Finally, this vaccination persona could benefit from more information specific to their beliefs about vaccines (e.g. the side effects of the vaccine as well as the longevity of protection offered by the vaccine).

**Table 4:** Vaccination status, sources of information and information needed to increase uptake of vaccines, female healthcare workers

	Vaccinated n (%)	Willing n (%)	Undecided n (%)	Not willing n (%)	p-value
Main source of information					
TV	89 (23.2)	8 (14.8)	21 (26.3)	32 (25.6)	0.401
Radio	129 (3.4)	1 (1.9)	4 (5.0)	5 (4.0)	0.761
Health staff	3279 (85.6)	47 (87.0)	62 (77.5)	81 (64.8)	< 0.001
Community health workers	419 (10.9)	6 (11.1)	6 (7.5)	8 (6.4)	0.320
Peers	462 (12.1)	2 (3.7)	10 (12.5)	5 (4.0)	0.011
Community leaders	105 (2.7)	2 (3.7)	4 (5.0)	1 (0.8)	0.322
Social Media	1407 (36.8)	12 (22.2)	26 (32.5)	53 (42.4)	0.065
SMS	182 (4.8)	1 (1.9)	1 (1.3)	2 (1.6)	0.123
Family	146 (3.8)	1 (1.9)	3 (3.8)	7 (5.6)	0.651
At work	766 (20.0)	6 (11.1)	25 (31.3)	26 (20.8)	0.030
Internet	754 (19.7)	10 (18.5)	14 (17.5)	14 (11.2)	0.122
Private doctors/clinics	611 (16.0)	6 (11.1)	19 (23.8)	21 (16.8)	0.206
No answer	1 (0.0)	0 (0.0)	0 (0.0)	2 (1.6)	< 0.001
**Most trusted source**					
TV	660 (17.2)	7 (13.0)	20 (25.0)	20 (16.0)	0.242
Radio	92 (2.4)	0 (0.0)	5 (6.3)	3 (2.4)	0.101
Health staff	3266 (85.3)	48 (88.9)	63 (78.8)	88 (70.4)	< 0.001
Community health workers	371 (9.7)	5 (9.3)	4 (5.0)	8 (7.2)	0.422
Peers	329 (8.6)	0 (0.0)	4 (5.0)	4 (3.2)	0.013
Community leaders	104 (2.7)	1 (1.9)	3 (3.8)	4 (3.2)	0.902
Social media	891 (23.3)	7 (13.0)	13 (16.3)	19 (15.2)	0.023
SMS	158 (4.1)	0 (0.0)	0 (0.0)	1 (0.8)	0.027
Family	101 (2.6)	2 (3.7)	2 (2.5)	1 (0.8)	0.596
Work	679 (17.7)	6 (11.1)	18 (22.5)	23 (18.4)	0.407
Other	4 (0.1)	0 (0.0)	0 (0.0)	1 (0.8)	0.175
Internet	544 (14.2)	4 (7.4)	9 (11.3)	10 (8.0)	0.097
Private doctors/clinics	828 (21.6)	11 (20.4)	28 (35.0)	38 (30.4)	0.004
No one	0 (0.0)	0 (0.0)	0 (0.0)	1 (0.)8	< 0.001
**Reasons for not trusting the vaccines**					
Confusing information	1346 (35.2)	22 (40.7)	39 (48.8)	66 (52.8)	< 0.001
Information not based on facts	580 (15.2)	5 (9.3)	10 (12.5)	22 (17.6)	0.480
Information not credible	508 (13.3)	3 (5.6)	9 (11.3)	914 (11.2)	0.329
Community doesn't trust the info	228 (6.0)	3 (5.6)	6 (7.5)	10 (8.0)	0.749
Local leaders don't trust the info	57 (1.5)	0 (0.0)	1 (1.3)	1 (0.8)	0.746
Don’t know	442 (11.5)	9 (16.7)	12 (15.0)	12 (9.6)	0.432
No answer	962 (25.1)	15 (25.9)	9 (11.3)	17 (13.6)	< 0.001
Other	3 (0.1)	0 (0.0)	0 (0.0)	0 (0.0)	0.956
I trust all	195 (5.1)	2 (3.7)	2 (2.5)	1 (0.8)	0.113
Rumours	2 (0.1)	0 (0.0)	0 (0.0)	0 (0.0)	0.988
**Information needed to increase the uptake of vaccines**					
How vaccines work	692 (18.1)	7 (13.0)	4 (5.0)	19 (15.2)	0.014
Who is eligible	287 (7.5)	5 (9.3)	3 (3.8)	8 (6.4)	0.561
What's inside the vaccines	471 (12.3)	6 (11.1)	10 (12.5)	16 (12.8)	0.992
What's the difference between different types	859 (22.44)	10 (18.5)	14 (17.5)	32 (25.6)	0.510
Side effects	1444 (37.7)	22 (40.7)	31 (38.8)	51 (40.8)	0.870
Longevity of protection	933 (24.4)	10 (18.5)	31 (38.8)	38 (30.4)	0.007
Effectiveness	785 (20.5)	11 (20.4)	18 (22.5)	25 (20.0)	0.975
Effectiveness against new strains	1229 (32.1)	11 (20.4)	26 (32.5)	29 (23.2)	0.053
Safety	1005 (26.3)	15 (27.8)	23 (28.8)	42 (33.6)	0.307
Types of vaccines	390 (10.2)	2 (3.7)	5 (6.3)	8 (6.)4	0.134
Stats about vaccines	7 (0.2)	0 (0.0)	0 (0.0)	0 (0.0)	0.924
How they work on pregnant women	0 (0.0)	0 (0.0)	0 (0.0)	0 (0.0)	0.000
Other	5 (0.1)	0 (0.0)	0 (0.0)	0 (0.0)	0.930
Don't know	61 (1.6)	0 (0.0)	0 (0.0)	1 (0.8)	0.450
No answer	111 (2.9)	2 (3.7)	3 (3.7)	8 (6.4)	0.404
Nothing	4 (0.1)	0 (0.0)	0 (0.0)	1 (0.8)	0.361
**Benefits of taking the Covid-19 vaccines**					
Feel protected	2178 (56.9)	27 (50.0)	22 (27.5)	21 (16.8)	< 0.001
Family and friends feel protected	1864 (48.7)	21 (38.9)	21 (26.3)	18 (14.4)	< 0.001
Motivated to do work	691 (18.1)	7 (13.0)	4 (5.0)	8 (6.4)	< 0.001
Motivated to socialize	482 (12.6)	5 (9.3)	2 (2.5)	3 (2.4)	< 0.001
Revert back to normal life	1058 (27.6)	18 (33.3)	14 (17.5)	9 (7.2)	< 0.001
Travel	1082 (28.3)	17 (31.5)	49 (61.3)	72 (57.6)	< 0.001
Less death	1697 (44.3)	20 (37.0)	20 (25.0)	13 (10.4)	< 0.001
Saving money	890 (23.2)	14 (25.9)	13 (16.3)	16 (12.8)	0.021

### Not vaccinated and unwilling

This vaccination persona is radically different compared to the other three. First, they tend to be less reliant on COVID-19 information. 10% of respondents who never receive information about COVID-19 tend to be unwilling to be vaccinated. Furthermore, this vaccination tends to have lower trust in the vaccines and significantly more negative beliefs regarding the COVID-19 vaccines, especially side effects. 15.7% of respondents who do not believe in the safety of the vaccines are also more likely to be vaccinated. 15.8% of respondents who believe that vaccines have low importance for one’s health, are also more likely to be unwilling to vaccinate.

This group has lower levels of trust in healthcare workers than other personas, and higher concerns about safety of vaccines. 40% of respondents who do not trust healthcare workers are also unwilling to vaccinate; 26.4% of respondents who do not trust any vaccine are also unwilling to receive the COVID-19 vaccine. They are more likely to be motivated by the opportunity to travel and less likely to recognise other benefits of vaccination such as protecting family and friends.

## Discussion

As indicated in the introduction, the aim of this paper was to provide a descriptive analysis of the four vaccination personas among female healthcare workers in Syria along three dimensions: demographic/socio-economic, attitude/beliefs around the vaccines and platforms that could be utilized to increase vaccination coverage, particularly among those who are vaccine hesitant. We observe a high rate of vaccination, with an overwhelming majority of female healthcare workers being vaccinated or willing to be vaccinated.

We find that the vast majority (93.7%) of female healthcare workers have received at least one COVID-19 vaccination dose. We ought to emphasize that our findings are in line with the existing evidence that stems from the context of low-income and fragile countries. A study based on Somalia for example, found that healthcare workers are more likely to be vaccinated, relative to the general population [18]. In addition, a study in the context of Yemen found that about 61.7% of healthcare workers agreed to accept a Covid-19 vaccine. The study also found that the strongest determinant of vaccine acceptance was access to vaccines [19]. Furthermore, in a review of existing evidence, Ghare et al. [20] also document a high uptake of COVID-19 vaccines, particularly among high income countries. Similarly high levels of vaccination among healthcare workers have also been documented in Ethiopia [21].

Consistent with existing evidence, we find that attitudes and beliefs around COVID-19 vaccines impact upon the decision to get a vaccination; more specifically, positive attitudes around effectiveness and safety of the vaccines increase the likelihood of being vaccinated or willing to be vaccinated. More specifically, healthcare workers which believe in the safety of the vaccines are twice as likely to get vaccinated relative to those who don’t. By contrast, we find that neutral attitudes regarding the vaccines are associated with vaccine indecision among female healthcare workers. Based on a literature review on the wider MENA region, Kacimi et al. (2022) find that concern about vaccine side effects and exigence for more efficacy and safety studies were the most commonly reported barrier and enabler for vaccine acceptance respectively. Perceived benefits associated with the vaccine were also found to be one of the main correlates of vaccine acceptance on sample of healthcare workers in Lebanon [22]. Beliefs around perceived safety of the vaccine was the main determinant of receiving a booster COVID-19 vaccine in a large sample of adults in the wider MENA region [23]. All of this evidence is consistent the main finding from our study—the attitudes and beliefs around the COVID-19 vaccine are the main predictor of the different vaccination personas presented in this paper.

Furthermore, the main finding of high vaccination rates among healthcare workers attests to the effectiveness of the priority afforded them in national policy, and also to the fact that healthcare workers have been at the forefront of the vaccination efforts. However, there are still some who are undecided about taking COVID-19 vaccines. This group tends to have neutral views and beliefs about the vaccines (on a scale from 1 to 5, they tend to respond with 3). Throughout the COVID-19 pandemic, misinformation was rife across the world, and healthcare workers may also have been affected by this, along with the general population. A study by Ali et al. [22] mentioned above, states that social norms are also one of the main correlates of the willingness to vaccinate against COVID-19. Similarly, the literature review by Kacimi et al. (2022) also highlights the importance of believing in conspiracy theories as one of the predictors of the willingness to receive a vaccine. Noting that they do have faith in their health colleagues, more formal professional networks for sharing information, supported by trusted and reliable leadership, may help to influence this group.

While campaigns tend to pay attention to the attitudes of the public towards vaccines, investing in capacity building activities which consider what healthcare workers themselves think and feel towards vaccines could play a key role in vaccination coverage. Evidence stemming from the Eastern Mediterranean region has also documented the link between vaccine beliefs and vaccination status. For example, in the Egyptian context, fear of side effects was associated with refusing the vaccines, while positive views around effectiveness and safety of COVID-19 vaccines was associated with higher uptake [9, 24]. The COVID-19 beliefs/vaccine uptake nexus was also confirmed in studies among healthcare workers in Sudan and Iraq [9, 24].

These findings provide solid basis for devising strategies for further tackling the vaccine hesitancy among the sub-population analysed in this paper. Previous research has mainly focused on tackling some of the individual barriers to vaccine uptake and has included, for example, peer outreach and increase in education resources among peers [25]. Addressing risk concerns, particularly among staff—particularly females of reproductive age—has also been documented as a positive practice [25]. An important aspect of these interventions is that they have been delivered by, inter alia, peers, which, as indicated in our survey is one of the most trusted sources of COVID-19 vaccine information among healthcare workers.

As in any research, this paper is also accompanied by some limitation. First, our analysis is descriptive in nature, and, in that respect, it cannot draw any causal links between vaccination status and variables of interest. Second, the survey is not representative of the entire population of female healthcare workers in Syria. Third, the survey was cross-sectional and provides a snapshot of vaccine uptake in Syria but does not enable analysis of differential rates of vaccine uptake. Fourth, the responses could be biased as they are self-reported. Finally, the sample is heavily biased towards vaccinated respondents, with a small number of responses in the other vaccination personas.

While this study showed very high vaccination among healthcare workers, even a small group of unvaccinated healthcare workers is important, given their risk and influence in the community. By further shedding light on some of the characteristics of different vaccination personas, this paper reinforces the need to tailor programs to different personas rather than a ‘one size fits all’ approach. Further research into these personas will certainly help to refine programming further.

## Supplementary Information

Additional file1 (DOCX 20 KB)

## Data Availability

No datasets were generated or analysed during the current study.

## Availability of data and materials

Available upon request.
