# Supplementary material for: Uptake of COVID-19 vaccine among female healthcare workers in Syria: results from a 2022 cross-sectional survey
Source: Confl Health. 2025 Aug 18;19:63. doi: 10.1186/s13031-025-00700-1 (PMC12363114; doi:10.1186/s13031-025-00700-1)
Supplement: Supplementary file 1 — Additional file1 (DOCX 20 KB) [file 13031_2025_700_MOESM1_ESM.docx]

**Appendix**

**Table A1. Logistic regression – correlates of vaccination status**

| vaccinated | Coef. | | St.Err. | t-value | | p-value | [95% Conf | | Interval] | Sig |
| --- | --- | --- | --- | --- | --- | --- | --- | --- | --- | --- |
| Age 18-55 (ref) | 1.000 | | . | . | | . | . | | . |  |
| Age over 55 | 0.822 | | 0.357 | -0.45 | | 0.652 | 0.351 | | 1.925 |  |
| Preparatory education (ref) | 1.000 | | . | . | | . | . | | . |  |
| Primary | 0.875 | | 0.459 | -0.26 | | 0.798 | 0.313 | | 2.445 |  |
| Secondary | 1.732 | | 0.367 | 2.59 | | 0.010 | 1.144 | | 2.623 | ** |
| University | 1.651 | | 0.364 | 2.27 | | 0.023 | 1.072 | | 2.543 | ** |
| News about Covid 19 – never (ref) | 1.000 | | . | . | | . | . | | . |  |
| Sometimes | 1.244 | | 0.371 | 0.73 | | 0.464 | 0.694 | | 2.232 |  |
| Often | 1.762 | | 0.594 | 1.68 | | 0.092 | 0.911 | | 3.410 | * |
| All the time | 6.156 | | 3.996 | 2.80 | | 0.005 | 1.725 | | 21.971 | *** |
|  | 1.000 | | . | . | | . | . | | . |  |
| I don’t trust vaccines (ref) | 1.000 | | . | . | | . | . | | . |  |
| I trust a few vaccines | 4.446 | | 1.031 | 6.43 | | 0.000 | 2.822 | | 7.004 | *** |
| I trust all vaccines | 11.352 | | 2.991 | 9.22 | | 0.000 | 6.774 | | 19.025 | *** |
| Challenges in obtaining a vaccine – 1 (ref) | 1.000 | | . | . | | . | . | | . |  |
| Challenges in obtaining a vaccine – 2 | 1.450 | | 0.401 | 1.34 | | 0.179 | 0.843 | | 2.494 |  |
| Challenges in obtaining a vaccine – 3 | 1.396 | | 0.388 | 1.20 | | 0.231 | 0.809 | | 2.407 |  |
| Challenges in obtaining a vaccine – 4 | 0.600 | | 0.218 | -1.41 | | 0.160 | 0.294 | | 1.223 |  |
| Challenges in obtaining a vaccine – 5 | 3.357 | | 1.794 | 2.27 | | 0.023 | 1.177 | | 9.571 | ** |
| Safety – 1 (ref) | 1.000 | | . | . | | . | . | | . |  |
| Safety – 2 | 0.525 | | 0.184 | -1.84 | | 0.065 | 0.264 | | 1.042 | * |
| Safety – 3 | 0.997 | | 0.351 | -0.01 | | 0.992 | 0.500 | | 1.986 |  |
| Safety – 4 | 2.926 | | 1.200 | 2.62 | | 0.009 | 1.309 | | 6.537 | *** |
| Safety - 5 | 2.337 | | 0.935 | 2.12 | | 0.034 | 1.067 | | 5.119 | ** |
| At risk of vaccine -1 (ref) | 1.000 | | . | . | | . | . | | . |  |
| At risk of vaccine -2 | 0.689 | | 0.231 | -1.11 | | 0.267 | 0.357 | | 1.330 |  |
| At risk of vaccine -3 | 1.271 | | 0.437 | 0.70 | | 0.485 | 0.648 | | 2.495 |  |
| At risk of vaccine 4 | 1.244 | | 0.483 | 0.56 | | 0.573 | 0.582 | | 2.662 |  |
| At risk of vaccine -5 | 4.180 | | 1.759 | 3.40 | | 0.001 | 1.832 | | 9.538 | *** |
| Constant | 0.379 | | 0.236 | -1.56 | | 0.120 | 0.112 | | 1.286 |  |
|  | | | | | | | | | | |
| Mean dependent var | | 0.950 | | | SD dependent var | | | 0.218 | |  |
| Pseudo r-squared | | 0.272 | | | Number of obs | | | 3813.000 | |  |
| Chi-square | | 300.203 | | | Prob > chi2 | | | 0.000 | |  |
| Akaike crit. (AIC) | | 1147.965 | | | Bayesian crit. (BIC) | | | 1297.873 | |  |
|  | | | | | | | | | | |
| **** p<0.01, ** p<0.05, * p<0.1. Challenges of obtaining a vaccine 1 – no challenge; 5 – very challenging; Safety of vaccines – 1 not safe; 5 very safe.* | | | | | | | | | |  |
